# Lessons From Recruitment to an Internet-Based Survey for Degenerative Cervical Myelopathy: Comparison of Free and Fee-Based Methods

**DOI:** 10.2196/resprot.6567

**Published:** 2018-02-05

**Authors:** Benjamin Davies, Mark Kotter

**Affiliations:** ^1^ Department of Academic Neurosurgery University of Cambridge Cambridge United Kingdom; ^2^ Anne McLaren Laboratory, Wellcome Trust - Medical Research Council Cambridge Stem Cell Institute, University of Cambridge Cambridge United Kingdom

**Keywords:** cervical, myelopathy, spondylosis, spondylotic, stenosis, disc herniation, ossification posterior longitudinal ligament, degeneration, disability, recovery, questionnaire, Internet survey, Google Adwords, advertising, social media, electronic survey, Internet, survey

## Abstract

**Background:**

Degenerative Cervical Myelopathy (DCM) is a syndrome of subacute cervical spinal cord compression due to spinal degeneration. Although DCM is thought to be common, many fundamental questions such as the natural history and epidemiology of DCM remain unknown. In order to answer these, access to a large cohort of patients with DCM is required. With its unrivalled and efficient reach, the Internet has become an attractive tool for medical research and may overcome these limitations in DCM. The most effective recruitment strategy, however, is unknown.

**Objective:**

To compare the efficacy of fee-based advertisement with alternative free recruitment strategies to a DCM Internet health survey.

**Methods:**

An Internet health survey (SurveyMonkey) accessed by a new DCM Internet platform (myelopathy.org) was created. Using multiple survey collectors and the website’s Google Analytics, the efficacy of fee-based recruitment strategies (Google AdWords) and free alternatives (including Facebook, Twitter, and myelopathy.org) were compared.

**Results:**

Overall, 760 surveys (513 [68%] fully completed) were accessed, 305 (40%) from fee-based strategies and 455 (60%) from free alternatives. Accounting for researcher time, fee-based strategies were more expensive ($7.8 per response compared to $3.8 per response for free alternatives) and identified a less motivated audience (Click-Through-Rate of 5% compared to 57% using free alternatives) but were more time efficient for the researcher (2 minutes per response compared to 16 minutes per response for free methods). Facebook was the most effective free strategy, providing 239 (31%) responses, where a single message to 4 existing communities yielded 133 (18%) responses within 7 days.

**Conclusions:**

The Internet can efficiently reach large numbers of patients. Free and fee-based recruitment strategies both have merits. Facebook communities are a rich resource for Internet researchers.

## Introduction

Degenerative Cervical Myelopathy (DCM) is a syndrome of cervical cord compression secondary to degenerative disease of the cervical spine [1]. Causative pathology includes disc herniation, osteophyte formation and ligament hypertrophy or ossification. Symptoms are often initially subtle and mislabelled as "old age."

Evidence from radiological series suggest 30%-59% of healthy individuals above the age of 50 show compression of the spinal cord on magnetic resonance imaging scans [2-4], of which up to 34% will develop myelopathy [5,6]. This equates to an estimated prevalence of 5% in patients over 50.

The epidemiology of DCM is poorly characterised at present, therefore such numbers are estimates [1]. The cause for this is multi-factorial but in part due to most research deriving from surgical studies [7], which only capture a sub-population of DCM patients. At present, not all DCM patients will reach specialist services and not all DCM patients will undergo surgery.

The Internet has become an attractive tool for medical research [8-10]. It enables access to individuals from across the globe who are of different ethnicities and socioeconomic backgrounds [11,12]. This access can be achieved in an unrivalled cost- and time-efficient manner. Therefore, for DCM, this presents an exciting opportunity to reach a larger, perhaps more complete, population and advance our understanding of the disease.

At present, most medical Internet research uses online questionnaires for data collection. Questionnaires are a research tool which can be completed by respondents anywhere at any time. Given the recognized significance of Patient Reported Outcome Measures [13], questionnaires, whether online or not, are a mainstay of clinical research.

Health surveys are notoriously difficult to recruit to [9]. Many different techniques have been trialled, including face-to-face events [11,12,14,15], print media [14-16], email [14,15,17,18], paid for Internet [14,18] or social media [12,15,16,18-22] advertising, social media engagement [11,15,17,18,21-25], and the use of incentives [17]. However, an optimum recruitment strategy has not yet emerged [9].

Comparing these studies to identify the most effective strategy is limited as many studies either do not provide a comparison arm or they bundle strategies together. Additionally, the implications of whether different strategies work across health research or are specific to certain conditions is unclear. Regardless, similar themes appear to be emerging; studies are moving away from print media or face-to-face strategies to Internet-based strategies [14,15,17]. Of these Internet strategies, Internet advertising specifically using Google AdWords or Facebook adverts, and/or social media engagement appear most popular and successful [12,20,22-24]. Google AdWords and Facebook Adverts are paid-for services whereas social media is free. A direct comparison of these methods has not been made specifically but cost-free options are clearly attractive if adequately effective.

Our overall objective, therefore, was to investigate whether the Internet could enable us to reach patients with DCM efficiently and on a large scale, and ask them about their disease and its consequences. In the absence of an ideal Internet recruitment strategy [9], we trialled fee-based methods (Google AdWords) and free methods (including social media and development of a condition specific website). We present a comparison of our experience and discuss their implications for others.

## Methods

Ethical approval for this survey was granted by the University of Cambridge (Cambridge, UK). The survey was hosted on Survey Monkey (SurveyMonkey, California, USA) and contained 38 questions.

### Tracking Recruitment Strategies

**Figure 1:**
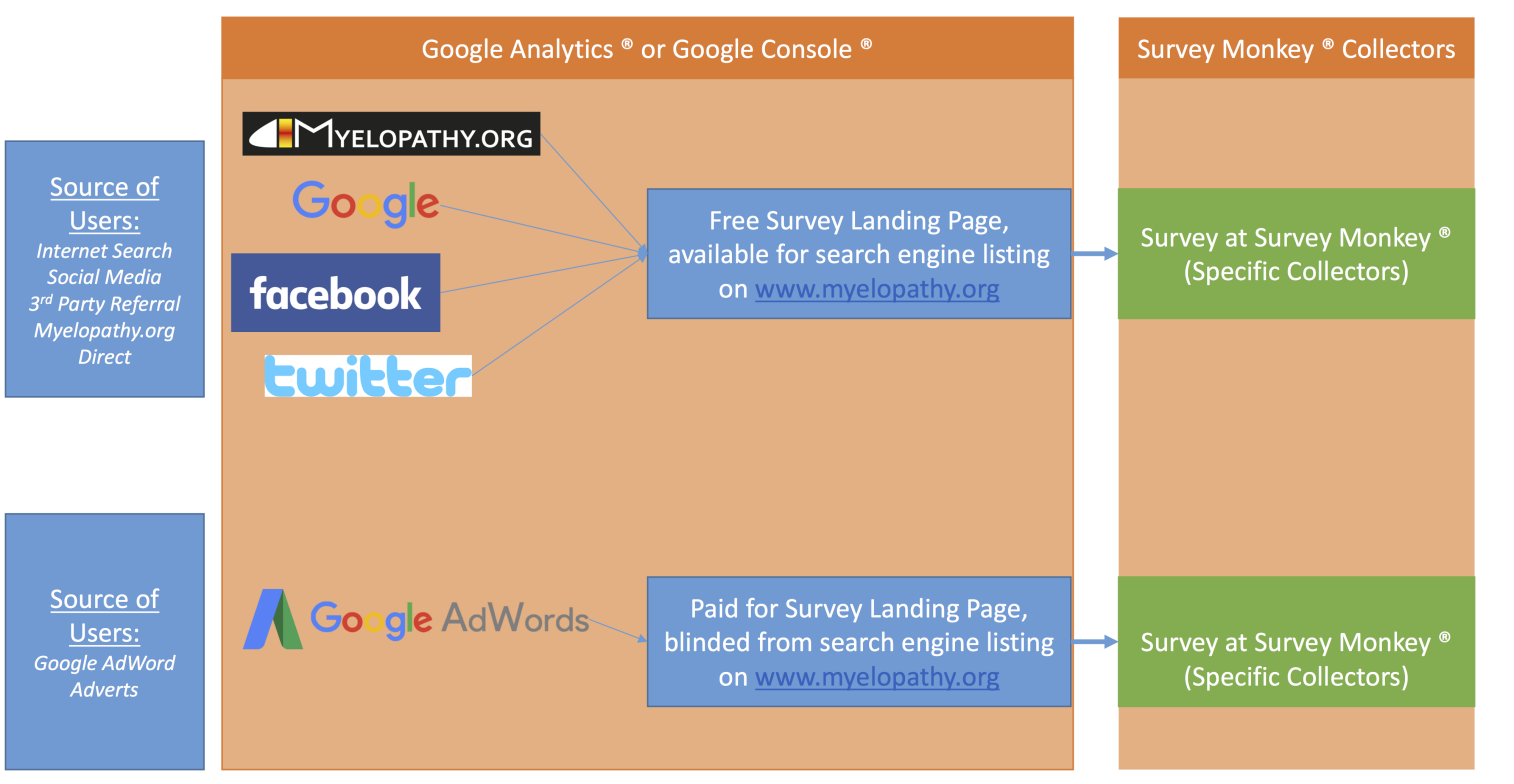
A flowchart describing the different access points (blue boxes) and flow to the survey, along with how they were tracked (orange boxes). Google AdWords adverts directed individuals to hidden landing page(s) at www.myelopathy.org, where interested viewers could click through to the survey at SurveyMonkey. All alternatives were directed to an open landing page and then to the survey. SurveyMonkey tracked respondents based on the URL used to access the survey. All prior data was tracked using Google Analytics and/or Google Console.

The SurveyMonkey advanced pro package was purchased to allow a single survey to have multiple collectors. A collector is a point of entry to the survey, which, in this study, was a custom URL. By using a different collector for each recruitment strategy, their individual performance could be tracked (Figure 1). These URLs were a combination of letters and numbers and were hidden from search engine listings, thus they were unlikely to be accessed directly.

Consequently, access to the survey was limited to landing pages hosted on our newly created DCM Internet platform, myelopathy.org. This enabled all traffic to be monitored precisely using Google Analytics (Google, California, USA). One landing page was accessible directly from the website and open to search engine listing. This was the portal for tracking free recruitment strategies. Google AdWords (Google, California, USA) adverts were directed to blind weblinks for myelopathy.org, hidden from search engine listing (Figure 1).

### Recruitment Strategies: Fee-Based Methods (Google AdWords)

Google advertising was capped at $15 per day. Other constant settings included using both the “Search Network” (appearing on the Google search engine) and the “Display Network” (appearing on third-party applications, through their embedded Google adverts), and allowing Google to optimize advert choice based on their overall performance (option “optimize for clicks”). Advertisement was limited to an English-speaking audience, prioritizing North America, Australia, and the UK.

**Figure 2:**
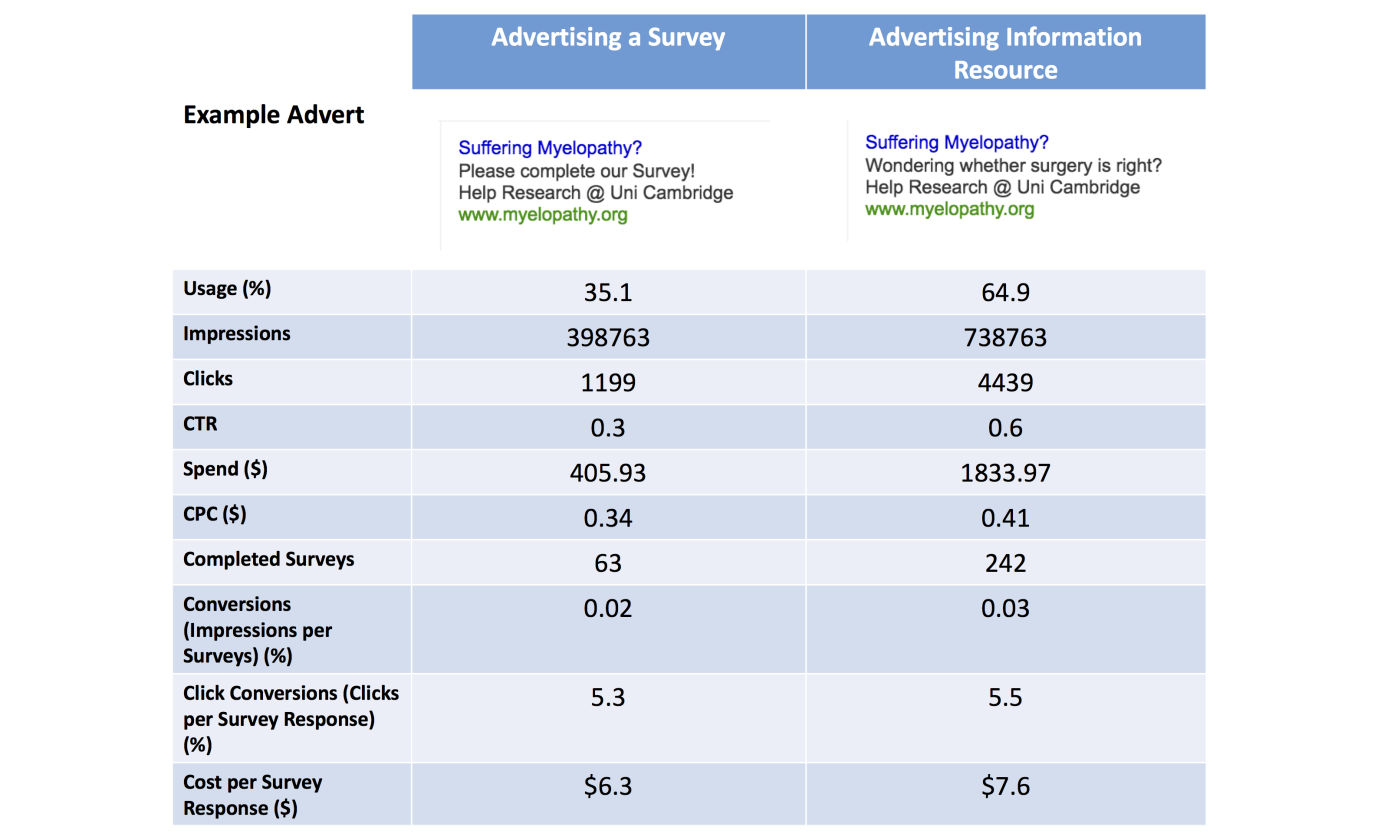
Comparison of different Google Advert types. One style advertised a clinical survey whilst another advertised information about surgery. Information about surgery was chosen, as surgery is the only treatment for Degenerative Cervical Myelopathy (DCM) and is the major focus of patient discussion following diagnosis. CTR: click-through-rate; CPC: cost-per-click.

Adverts were created within a single group so that they would be displayed against all chosen keywords. Two adverts were developed using Google AdWord advice pages only (Figure 2). The research team had no prior experience with online advertising. Themes taken from these advice pages included using a relevant keyword within the title (“myelopathy”), identifying who you are (“Uni Cambridge” and “www.myelopathy.org”), what you are offering and what makes you unique (“Help Research”). With regards to this latter aspect, the only variations were whether “Please complete our survey” or “Wondering whether surgery is right?” were used. These adverts remained unchanged throughout the period of Google AdWord advertising for comparison.

Keywords were author-selected based on their theoretical relevance. Google Trends (Google, California, USA) was used to suggest related search terms. Chosen keywords were adapted and refined based on performance metrics.

### Recruitment Strategies: Free Methods

#### Website

The website (www.myelopathy.org) was developed using Weebly (Weebly, San Francisco, USA). The intended target audience was patients, so initial content was produced to explain the disease, its symptoms, and its treatments. Each week further content was added, including further generic content, as well as expert articles, patient stories, and blog entries concerning the latest research. The home page opened with an advert for the survey and additional content included links to the survey.

The website was submitted to Google Search Console (Google, California, USA) for indexing, which was completed November 2, 2015. Alongside Google Analytics, Google Search Console was used to track the website's overall performance in Google search returns. This is a relatively new service and data is only available from May 2016.

#### Social Media

Along with the website, a Twitter (Twitter, California, USA) account (@myelopathyorg) and a Facebook (Facebook, California, USA) page (www.facebook.com/myelopathy) were created. Website content was promoted, including postings and tweets using these accounts.

In addition, active related Twitter users/organizations (such as charities) and Facebook groups were approached to advertise the survey. Users, organizations, or Facebook groups were targeted a maximum of two times, at least 1 month apart. Facebook groups were approached using a researcher’s personal Facebook profile, ensuring their privacy settings were optimized (as Facebook does not permit an organization to directly join groups). Permission from group administrators was sought before posting in closed Facebook Groups. The resultant post was made either by the researcher or the group administrator depending on their preference.

Potential Facebook groups or organizations and Twitter users or organizations were identified by searching the platforms for “myelopathy” and related disease specific terms. Initial approaches were simply as informal tweets (Twitter) or direct messages (Facebook and Twitter). No specific template was used. Social media links were embedded with Urchin Tracking Module codes to allow Google Analytics to register their impact individually.

#### Third-Party Websites

Related online content providers were approached to reference myelopathy.org. Google and Yahoo email indexes were not explored given the poor response by Morgan et al (2013)[18].

### Study Structure and Analysis

Myelopathy.org went live October 1, 2015. Recruitment strategies commenced November 2, 2015. The initial four-week period was used as a control period to test the platform, register with Google Search Console, and measure any preintervention traffic. The total available budget for Google AdWords was $2200 and this was continued until this budget was exhausted.

The principal outcome to evaluate recruitment strategies was the number of survey responses. Complete survey responses (ie, questionnaires answered in full) were differentiated from incomplete responses. The completion rate was the proportion of fully completed survey responses.

Metrics of Google AdWord advert performance included impressions (number of times the advert or link was displayed), clicks (number of times the advert or link was clicked on), click-through-rate (CTR) (proportion of clicks to impressions), total cost, and cost-per-click (CPC) (cost per advert clicked on).

Metrics of user activity, tracked via Google Analytics, included views (number of times a page was viewed), unique visitor views (number of times a page was viewed by different users), and bounce rate (proportion of times a user came directly to a page and then left the website without clicking on any additional links).

Individualised SurveyMonkey collectors allowed the number of responses per intervention to be tracked directly, as the Google Analytics could not assess whether a survey was completed or not. Figure 1 provides an overview of how the free and fee-based recruitment strategies were tracked. The time spent managing interventions by the researcher between week 8 and 12 was logged per action.

## Results

**Figure 3:**
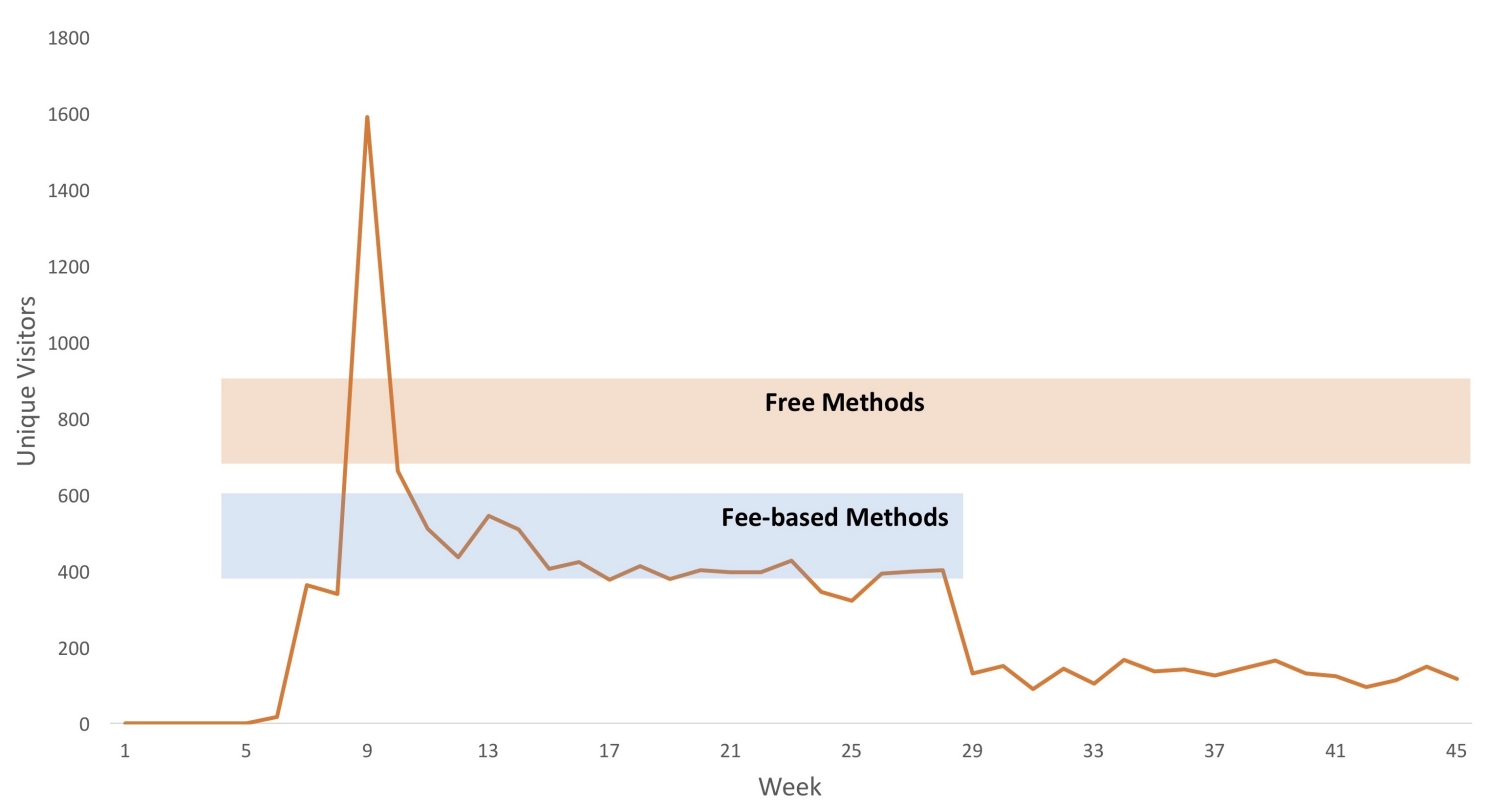
Unique visitors to Myelopathy.org. Active survey recruitment started during week 6 of this study. Google AdWords was used between week 6 and week 28 (blue bar) compared to free recruitment methods which continued throughout (orange bar).

As outlined, myelopathy.org went live on October 1, 2015 and the recruitment interventions commenced following a 1-month control period. In total 12,671 unique users have accessed the platform, rising from 0 per week to an average of 454 per week using both fee and free methods, settling at an average of 130 per week with just free methods continuing (Figure 3). Excluding an outlier in week 9, during these two periods, traffic was very consistent; both periods having a relative standard deviation of 17%.

Overall, 760 surveys were completed during the 10-month period of which 513 (68%) were fully completed.

### Fee-Based Methods: Google AdWords

Google AdWords was used for 22 weeks (Week 6 -28) at a total cost of $2239.90 and resulted in 5638 users who clicked on adverts, at a CPC of $0.4. Using the fee-based method SurveyMonkey collectors, 305 (CTR 5%) surveys were completed, accessed through Google AdWords 98.5% of the time, of which 195 (64%) of the survey responses were complete.

Adverts attached to Google AdWords were broadly split into two categories; those advertising the DCM health survey and those advertising myelopathy.org as a DCM information resource (Figure 2). Adverts specifically inviting users to participate in a survey (CTR 3%) were less likely to be clicked on than adverts promoting myelopathy.org as an information resource (CTR 6%) (Figure 2). Consequently, Google’s “Optimize for Clicks” algorithm favored their presentation. However, the bounce rate for users arriving at the survey page expecting information was higher (90% vs 50%) than those having clicked through for a survey. Despite this, there was an equivocal completion rate of 5.3% and 5.5%, and the survey specific adverts were financially more efficient at a cost of $6.30 per response compared to $7.60 per response.

AdWords were chosen with the help of Google Trends and refined based on performance. In total over 100 keyword combinations were tried. These were typically related to “myelopathy,” the causative pathologies, or treatment. The most effective keywords and eventual focus of Google AdWords were terms related to understanding myelopathy, eg, “what is cervical myelopathy?” and “cervical myelopathy symptoms and treatment.” There was no relationship between the cost of an advert and its likelihood to yield a survey response. Of the advertisements, 46.7% were placed independently using the Google Display Network, ie, appearing as third-party advertisement on websites it considered related. These adverts performed better than those appearing on Google Search with a lower cost per click ($0.30 versus $0.48) and bounce rate (73% versus 90%). Unfortunately, our analytics design could not differentiate survey responses between these groups.

### Free Methods

**Figure 4:**
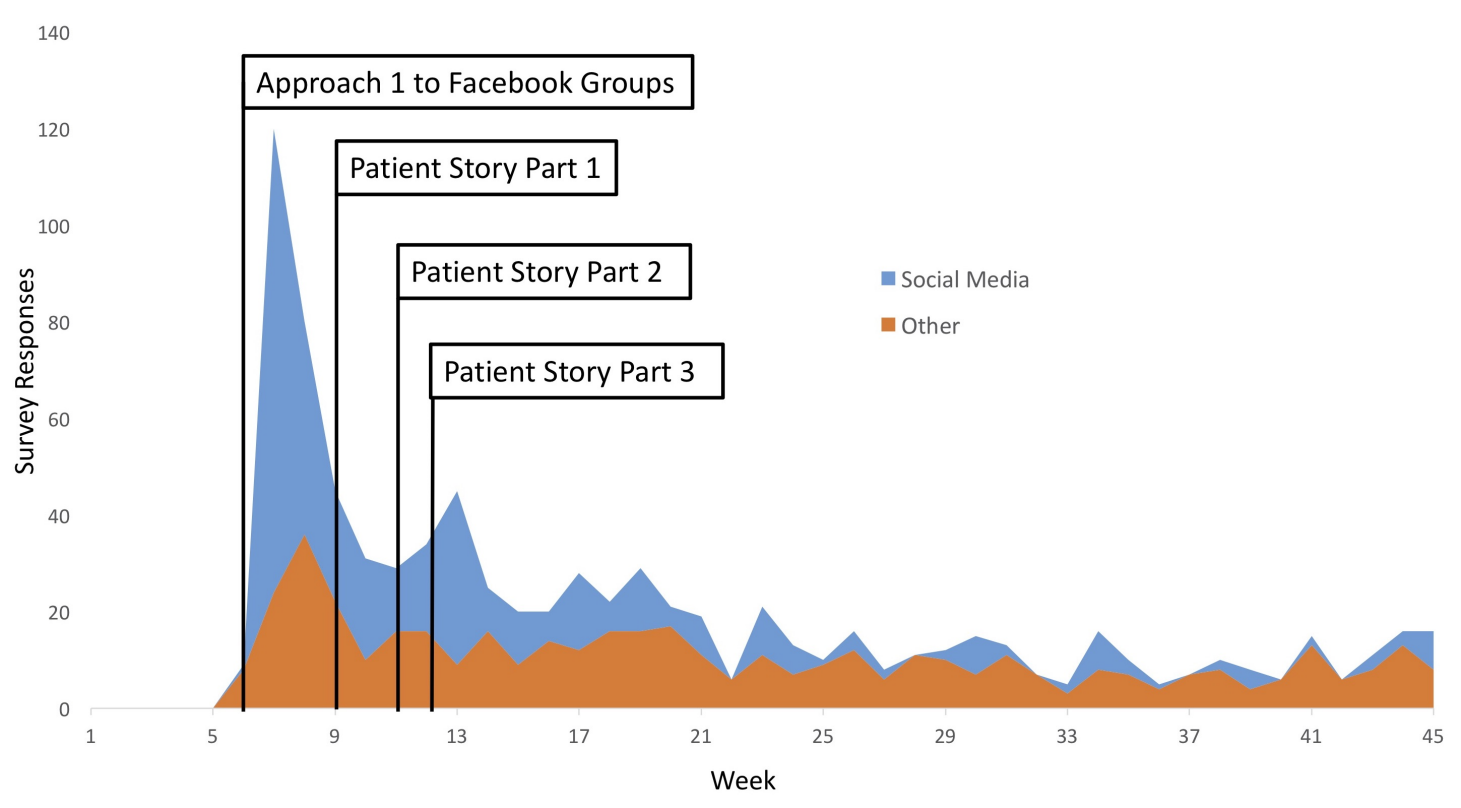
Survey traffic from free recruitment methods, including examples of successful interventions. Social Media (blue) was particularly effective in the early stages.

The survey landing page for free methods was accessed 730 times. This generated 455 survey responses (CTR 62%), of which 312 (69%) were complete. Of these viewers, 40 (5.5%) came from Google AdWords, whereas the majority came from social media (249 [55%]), most notably Facebook (239 [53%]) (Figure 4). Users arriving at the landing page from Facebook had a CTR of 73%, compared to 45% for Twitter.

We identified 9 related patient support groups on Facebook whose membership numbers ranged from 33 to 2137. However, only 4 of these groups appeared to be active. Of the active groups, combined membership exceeded 4000 people. Within 7 days of the initial approach to these groups during week 5, 133 users had accessed and completed the survey. Repeat approaches in week 10 had less significant impacts (Figure 4).

The sharing of new website content on social media, particularly Facebook, was beneficial. Our opening piece, a story of a patient (identified from the active Facebook communities) drew significant attention. It was shared 176 times in 12 hours and lead to our peak website traffic in week 9 of 1591 unique users (Figure 4). However, this story had not been linked to the survey and, as such, only 25 of these users accessed the survey (yielding 3 survey responses). By advertising the survey within subsequent articles, more survey responses were gained even though the articles generated less traffic.

**Table 1:** Comparison of Free and Fee-based recruitment methods, data from week 6 to 28.

Variable		Fee-based methods (Google AdWords)	Free methods
**Survey Responses**		305	367
	Complete n (%)	195 (64)	250 (68)
Advertising Cost/Response ($)		7.40	0
Click-through-rate (%)		5	57
Time/Response Estimate (mins)		2	16
Total Cost/Response Estimate ($)		7.80	3.20

Twitter was less fruitful in identifying and engaging participants. No existing specific organization or discussion thread or related hashtag were identified. Based on their potential relevance, 22 charities were approached. Of these, 11 (50%) retweeted the survey, yielding 9 survey responses. Keyword searches identified 6 individual users, of which 1 responded to the survey.

Third-party websites also provide a small number of respondents. These websites referred to myelopathy.org as an information source and not the survey page specifically. NHS Choices (www.nhs.uk), where myelopathy.org is listed as a DCM support organization, provided 615 unique user referrals of which 6 (0.1%) completed the survey. Wikipedia provided 70 unique user referrals but no survey responses.

### Comparison of Fee-Based and Free Methods

From week 6 to 28 both fee-based and free recruitment methods were used. During this time, free recruitment methods returned 367 survey responses. When directly compared (Table 1), in addition to being free, these methods were more likely to yield a response from the targeted audience (CTR 57% versus 5%). Following initial setup, between week 8 and 12, all activities related to either method were time-logged. In these 4 weeks, free recruitment methods required 10 hours and 21minutes of maintenance by a researcher, compared to just 30 minutes for the Google AdWords. Based on survey responses during these 4 weeks, free methods were estimated to require 16 minutes per survey response compared to 2 minutes for Google AdWords. Using a UK junior postdoctoral research salary of $26,000 to estimate the administrative costs, free recruitment strategies yielded responses at a cost of $3.17 per response, compared to $7.80 for fee-based methods.

## Discussion

Over 700 patients with DCM were recruited to an Internet survey in 10 months. This places it amongst the largest clinical DCM data sets based on sample size [7]. Simple, cost-free techniques, particularly approaching Facebook groups, were effective in reaching a motivated audience. However, this was time-consuming and may have a saturation point. Google AdWords was an effective and time-efficient alternative, but its use comes with a price.

### Limitations

From the outset, it is important to acknowledge the limitations of this study. This was an adaptive study design, more akin to a quality improvement process. Interventions, therefore, were not prescriptively performed throughout the period and it is not possible to directly compare these individually any further. For example, it is likely Facebook activity targeted the same users on each occasion, therefore the sequence in which interventions occurred will no doubt have contributed to their specific impact, but this cannot be commented on further.

Additionally, Google Analytics shows that fee-based and free collectors had small overlaps. Given the complex URLs for fee-based collectors and their blinding to search engines, it is very unlikely access to the survey could come about by any other means than clicking on the Google AdWord advert. Therefore, for fee-based methods, this is unlikely to be significant. These recorded accesses are instead, most likely to represent maintenance views by the research team and the method of access (direct or via Weebly) on Google Analytics would reflect this. However, for free methods collectors, users may have initially reached the platform via a Google AdWord advert and explored the platform further, before deciding to participate in the survey. Therefore, this may have yielded some survey responses. However, the maximum this would be is 40 responses (assuming 100% completed a survey) and this would not alter the overall conclusions.

### Findings in Context

Our findings further demonstrate the power of the Internet to reach patients, either by paid advertisement or free alternatives. At present, no absolute strategy has emerged as the most successful [8,9], and it is likely that any strategy needs to be considered in the context of the individual project as both strategies have their merits. Most recent examples have used a combination of paid advertising and alternative methods, including social media [15,21,22].

In the first quarter of 2016, Google and Facebook held 85% of the global digital advertising market [26]. Their popularity is also reflected in recent recruitment studies, particularly Facebook [8]. The attraction of Facebook advertising for researchers is the ability to specify target demographics. This has been effectively utilised, for example, in sexual health studies [21]. However, Facebook adverts only target the Facebook community. In this study we chose Google AdWords because the demographics of our patients are not so clearly defined and potentially include older individuals less likely to be using Facebook [27]. We also wanted to avoid overlap with our alternative Facebook interventions. This overlap may explain the contrasting findings of Yuan et al (2014) in their recruitment of HIV positive patients to an Internet survey. Their study used very similar recruitment strategies, with the exception that their paid advertising was conducted on Facebook as opposed to Google AdWords. They found only a very weak correlation between social media engagement and survey responses, concluding it was less efficient than Facebook advertising [21]. Likewise, Valdez et al (2014) showed promising, albeit less significant, engagement from social media groups.

Therefore, the significant impact of social media engagement here is a novel finding. This success may stem from several unmeasured factors. Firstly, compared to the very simple one sentence of text allowed by Google AdWords, posts to Facebook could contain a far more detailed overview of the study and its objectives. This may have helped capture an audience and explain their greater motivation to complete the survey fully having accessed it. Additionally, our modifications to Google AdWords were very basic and our relative inexperience with this tool undoubtedly had some influence. Furthermore, the use of Facebook groups led to patient support and promotion of the survey. For example, the Facebook posts often developed into conversation threads, with group users commenting when they had responded. This maintained the post’s prominence within the group for some time. This promotion by the users themselves may also explain the similar trends in the efficacy of social media and other free advertising strategies (Figure 4) in weeks 5-9.

In DCM, the Facebook communities are relatively small which may limit their representation and overall number of responses [23]. However, for many conditions this is not the case. A simple search of ‘Multiple Sclerosis Group’ in Facebook returns many groups, with the top four groups have a combined membership of >22,000. Many alternative free recruitment strategies have been tried, including email, alternative social media, and alternative third-party websites, but as with our findings, their impact has been relatively minor [9,15,21,23,28].

### Conclusions

A large number of patients can be efficiently reached using the Internet. Internet advertisements and free alternatives both have their merits. Google AdWords provides a simple and constant stream of traffic, although comes with significant cost. The targeting of existing communities was cheaper and identified a more motivated user. Whilst this exposes the researcher’s identity, this is a highly effective and simple strategy.

## Abbreviations

CPC: cost-per-click
CTR: click-through-rate
DCM: Degenerative Cervical Myelopathy

## References

[1] Nouri A, Tetreault L, Singh A, Karadimas SK, Fehlings MG (2015). Degenerative Cervical Myelopathy: Epidemiology, Genetics, and Pathogenesis. Spine.

[2] Okada E, Matsumoto M, Ichihara D, Chiba K, Toyama Y, Fujiwara H, Momoshima S, Nishiwaki Y, Hashimoto T, Ogawa J, Watanabe M, Takahata T (2009). Aging of the cervical spine in healthy volunteers: a 10-year longitudinal magnetic resonance imaging study. Spine.

[3] Kovalova I, Kerkovsky M, Kadanka Z, Kadanka Z, Nemec M, Jurova B, Dusek L, Jarkovsky J, Bednarik J (2016). Prevalence and Imaging Characteristics of Nonmyelopathic and Myelopathic Spondylotic Cervical Cord Compression. Spine.

[4] Nakashima H, Yukawa Y, Suda K, Yamagata M, Ueta T, Kato F (2015). Abnormal findings on magnetic resonance images of the cervical spines in 1211 asymptomatic subjects. Spine.

[5] Wilson JR, Barry S, Fischer DJ, Skelly AC, Arnold PM, Riew KD, Shaffrey CI, Traynelis VC, Fehlings MG (2013). Frequency, timing, and predictors of neurological dysfunction in the nonmyelopathic patient with cervical spinal cord compression, canal stenosis, and/or ossification of the posterior longitudinal ligament. Spine.

[6] Bednarik J, Kadanka Z, Dusek L, Kerkovsky M, Vohanka S, Novotny O, Urbanek I, Kratochvilova D (2008). Presymptomatic spondylotic cervical myelopathy: an updated predictive model. Eur Spine J.

[7] Davies BM, McHugh M, Elgheriani A, Kolias AG, Tetreault LA, Hutchinson PJA, Fehlings MG, Kotter MRN (2016). Reported Outcome Measures in Degenerative Cervical Myelopathy: A Systematic Review. PLoS One.

[8] Lane TS, Armin J, Gordon JS (2015). Online Recruitment Methods for Web-Based and Mobile Health Studies: A Review of the Literature. J Med Internet Res.

[9] Edwards PJ, Roberts I, Clarke MJ, Diguiseppi C, Wentz R, Kwan I, Cooper R, Felix LM, Pratap S (2009). Methods to increase response to postal and electronic questionnaires. Cochrane Database Syst Rev.

[10] Brueton VC, Tierney J, Stenning S, Harding S, Meredith S, Nazareth I, Rait G (2013). Strategies to improve retention in randomised trials. Cochrane Database Syst Rev.

[11] Close S, Smaldone A, Fennoy I, Reame N, Grey M (2013). Using Information Technology and Social Networking for Recruitment of Research Participants: Experience From an Exploratory Study of Pediatric Klinefelter Syndrome. J Med Internet Res.

[12] Admon L, Haefner JK, Kolenic GE, Chang T, Davis MM, Moniz MH (2016). Recruiting Pregnant Patients for Survey Research: A Head to Head Comparison of Social Media-Based Versus Clinic-Based Approaches. J Med Internet Res.

[13] Macefield RC, Jacobs M, Korfage IJ, Nicklin J, Whistance RN, Brookes ST, Sprangers MAG, Blazeby JM (2014). Developing core outcomes sets: methods for identifying and including patient-reported outcomes (PROs). Trials.

[14] Wong CK, Horn-Ross PL, Gee GC, Shariff-Marco S, Quach T, Allen L, Bautista R, La CPQ, Tseng W, Chang P, Clarke CA, Yang J, Le GM, Canchola A, Irwin ML, Lee SS, Gomez SL (2016). Strategies for recruiting representative samples of Asian Americans for a population-based case-control study. J Epidemiol Community Health.

[15] Harris ML, Loxton D, Wigginton B, Lucke JC (2015). Recruiting online: lessons from a longitudinal survey of contraception and pregnancy intentions of young Australian women. Am J Epidemiol.

[16] Carter-Harris L, Bartlett ER, Warrick A, Rawl S (2016). Beyond Traditional Newspaper Advertisement: Leveraging Facebook-Targeted Advertisement to Recruit Long-Term Smokers for Research. J Med Internet Res.

[17] Koo M, Skinner H (2005). Challenges of internet recruitment: a case study with disappointing results. J Med Internet Res.

[18] Morgan AJ, Jorm AF, Mackinnon AJ (2013). Internet-based recruitment to a depression prevention intervention: lessons from the Mood Memos study. J Med Internet Res.

[19] Crosier BS, Brian RM, Ben-Zeev D (2016). Using Facebook to Reach People Who Experience Auditory Hallucinations. J Med Internet Res.

[20] Bold KW, Hanrahan TH, O'Malley SS, Fucito LM (2016). Exploring the Utility of Web-Based Social Media Advertising to Recruit Adult Heavy-Drinking Smokers for Treatment. J Med Internet Res.

[21] Yuan P, Bare MG, Johnson MO, Saberi P (2014). Using online social media for recruitment of human immunodeficiency virus-positive participants: a cross-sectional survey. J Med Internet Res.

[22] Amon KL, Campbell AJ, Hawke C, Steinbeck K (2014). Facebook as a recruitment tool for adolescent health research: a systematic review. Acad Pediatr.

[23] Valdez RS, Guterbock TM, Thompson MJ, Reilly JD, Menefee HK, Bennici MS, Williams IC, Rexrode DL (2014). Beyond traditional advertisements: leveraging Facebook's social structures for research recruitment. J Med Internet Res.

[24] O'Connor A, Jackson L, Goldsmith L, Skirton H (2014). Can I get a retweet please? Health research recruitment and the Twittersphere. J Adv Nurs.

[25] Khatri C, Chapman SJ, Glasbey J, Kelly M, Nepogodiev D, Bhangu A, Fitzgerald JE, STARSurg C (2015). Social media and internet driven study recruitment: evaluating a new model for promoting collaborator engagement and participation. PLoS One.

[26] Garrahan M (2016). Advertising: Facebook and Google build a duopoly. Financial Times.

[27] Davies BM, McHugh M, Elgheriani A, Kolias AG, Tetreault L, Hutchinson PJA, Fehlings MG, Kotter MRN (2017). The reporting of study and population characteristics in degenerative cervical myelopathy: A systematic review. PLoS One.

[28] Treweek S, Pitkethly M, Cook J, Kjeldstrøm M, Taskila T, Johansen M, Sullivan F, Wilson S, Jackson C, Jones R, Mitchell E (2010). Strategies to improve recruitment to randomised controlled trials. Cochrane Database Syst Rev.

